# Imatinib induces apoptosis by inhibiting PDGF- but not insulin-induced PI 3-kinase/Akt survival signaling in RGC-5 retinal ganglion cells

**Published:** 2009-08-15

**Authors:** Swarajit K. Biswas, Yan Zhao, Lakshman Sandirasegarane

**Affiliations:** 1 Penn State Hershey Heart & Vascular Institute, Department of Medicine, Penn State College of Medicine, Hershey, PA; 2 Department of Pharmacology, Penn State College of Medicine, Hershey, PA

## Abstract

**Purpose:**

Platelet-derived growth factor (PDGF) and insulin promote the survival of neuronal cells, including retinal ganglion cells (RGCs), via activation of phosphoinositide 3-kinase (PI 3-kinase)/Akt signaling. Of importance, recent studies have shown that imatinib inhibition of PDGF receptors induces retinal toxicity in some patients. To date, the extent of activation and the functional significance of insulin-induced PI 3-kinase/Akt signaling remain unclear in the context of dysregulated PDGF receptor signaling in retinal cells. In the present study, we tested the hypothesis that the pro-survival effect of insulin-induced PI 3-kinase/Akt signaling is compromised by imatinib inhibition of PDGF receptor signaling in RGCs.

**Methods:**

RGC-5 cells were subjected to acute and long-term treatments with imatinib, a PDGF receptor tyrosine kinase inhibitor. Afterwards, the changes in RGC phenotype and apoptotic markers were assessed by fluorescence and phase contrast microscopy and caspase-3/poly(ADP-ribose) polymerase (PARP) cleavage, respectively. In addition, imatinib regulation of PDGF- and insulin-induced PI 3-kinase/Akt survival signaling was determined by immunoblot analyses, immunoprecipitation, and in vitro PI 3-kinase assays.

**Results:**

Treatment of RGC-5 cells with imatinib for up to 48 h resulted in apoptosis, which was not rescued by insulin supplementation. The apoptotic phenotype was associated with upregulation of cleaved caspase-3 and cleaved poly(ADP-ribose) polymerase. Time dependency experiments revealed that imatinib-mediated apoptosis was preceded by early and sustained abrogation of PDGF-induced increases in PDGF receptor tyrosine phosphorylation and phosphotyrosine-associated PI 3-kinase activity. In addition, imatinib inhibited PDGF-induced downstream phosphorylation of Akt, GSK-3β, and p70S6kinase. However, imatinib exposure did not affect insulin-induced insulin receptor substrate (IRS)-associated PI 3-kinase activity and the downstream phosphorylation of Akt, GSK-3β, and p70S6kinase.

**Conclusions:**

Together, these data indicate that disruption of PDGF receptor signaling compromises the pro-survival effect of insulin-induced IRS-dependent PI 3-kinase/Akt signaling in RGCs, and that the maintenance of PDGF-induced PI 3-kinase/Akt signaling is critical for the survival of retinal neuronal cells.

## Introduction

The central nervous system consists of different neuronal cell types, including retinal, cerebellar, and cortical neuronal cells [1], the survival of which depends in part on insulin receptor signaling [2-9]. Impaired insulin receptor stimulation of survival signals such as insulin receptor substrate (IRS)-associated phosphoinositide 3-kinase (PI 3-kinase) and Akt results in caspase-3 activation and apoptotic cell death in neurons [4,6,10]. Genetic ablation of rod photoreceptor-specific insulin receptors in mouse retinas results in light-induced photoreceptor degeneration, which is characterized by diminished PI 3-kinase/Akt activation [6]. In addition, deficiency of insulin receptor substrate-2 (IRS-2) in mice induces ganglion cell and photoreceptor loss, associated with diminished Akt activation and increased caspase-3 activation [11]. In brain/neuron-specific insulin receptor knockout mice, insulin cannot induce IRS-associated PI 3-kinase activity and Akt phosphorylation to prevent neuronal cell apoptosis [9]. These studies clearly demonstrate that the maintenance of functional insulin receptor survival signaling is pivotal to the viability of postmitotic neurons.

Platelet-derived growth factor (PDGF) is another important neurotrophin that promotes the survival of central nervous system neurons, including retinal ganglion cells (RGCs). In this regard, PDGF induces PI 3-kinase/Akt survival signaling to prevent apoptotic cell death [12-15]. Transection of the optic nerve is associated with RGC apoptosis, and this has been attributed to diminished expression levels of PDGF [16,17]. Mouse brains deficient in neuronal PDGF receptor-β are vulnerable to cerebral damage as revealed by increased neuronal cell death in response to N-methyl-D-aspartate [18]. These studies suggest that the maintenance of functional PDGF receptor signaling is also important in promoting neuronal cell survival.

Recent studies have shown that imatinib inhibition of PDGF receptors induces retinal toxicity in patients with chronic myeloid leukemia (CML) [19-23]. These observations suggest that imatinib treatment would affect PDGF-induced PI 3-kinase/Akt survival signaling, which however has not been examined in retinal cells, including RGCs. Furthermore, imatinib therapy in CML patients with diabetes improves insulin sensitivity and fasting blood glucose levels [24,25]. These findings raise the possibility that imatinib inhibition of PDGF receptors may enhance insulin-induced pro-survival signaling. On the contrary, recent studies with hepatoma cells have shown that imatinib attenuates insulin-induced phosphorylation of Akt and glycogen synthase kinase-3β (GSK-3β) [26]. Hence, it is important to compare the effects of imatinib on PDGF-versus insulin-induced PI 3-kinase/Akt signaling and retinal cell survival. In the present study, we tested the hypothesis that the pro-survival effect of insulin-induced PI 3-kinase/Akt signaling is compromised by imatinib inhibition of PDGF receptor signaling in RGCs.

It is widely known that insulin and PDGF activate PI 3-kinase via IRS-dependent and IRS-independent mechanisms, respectively [12,27,28]. Insulin receptor stimulation promotes tyrosine phosphorylation of insulin receptor substrates (IRS-1 and IRS-2) to recruit p85 regulatory subunits of PI 3-kinase, whereas PDGF promotes PDGF receptor tyrosine phosphorylation to recruit p85 regulatory subunits. The recruitment of p85 regulatory subunit leads to a conformational change in the p110 catalytic subunit to activate PI 3-kinase. The isoforms of p85 regulatory subunit include p85α, p85β, and the splice variants (p55γ, p55α, and p50α) and those of p110 catalytic subunit include p110α, p110β, and p110δ, and they belong to class I_A_ PI 3-kinase [29]. Importantly, class I_A_ PI 3-kinase activation constitutes a major source for the generation of different phosphatidylinositol 3,4,5-trisphosphate (PIP3) lipid species [29,30]. Insulin or PDGF stimulation of class I_A_ PI 3-kinase enhances cellular levels of PIP3, which results in the phosphorylation of downstream effectors, including Akt, glycogen synthase kinase-3β (GSK-3β), and p70S6kinase. To date, insulin and PDGF stimulation of retinal cell class I_A_ PI 3-kinase and its downstream effectors have not been examined in parallel, particularly in the context of dysregulated PDGF receptor signaling.

To determine the functional consequences of impaired PDGF receptor signaling on neuronal cell phenotype, we exposed RGC-5 retinal neuronal cells in culture to imatinib, a PDGF receptor tyrosine kinase inhibitor. After acute and long-term treatments with imatinib, we examined the changes in RGC phenotype and the associated changes in apoptotic markers. In addition, we performed a detailed analysis of PI 3-kinase/Akt survival signaling by determining PDGF- and insulin-induced changes in PI 3-kinase activity and the phosphorylation of downstream effectors such as Akt, GSK-3β, and p70S6kinase.

## Methods

### Materials

Recombinant human platelet-derived growth factor-BB (PDGF-BB) was purchased from R&D Systems (Minneapolis, MN). Human insulin (Novolin R) and imatinib mesylate (Gleevec^®^) were obtained from Hershey Medical Center Pharmacy (Hershey, PA). The primary antibodies for PDGFR-β (28E1), phospho-PDGFR β (Tyr751), phospho-IGF-1 receptor/insulin receptor, phospho-Akt (Ser473), Akt, phospho-GSK-3β, phospho-p70S6kinase (Thr421/Ser424), caspase-3, and poly(ADP-ribose) polymerase (PARP) were purchased from Cell Signaling (Beverly, MA). The primary antibodies for phosphotyrosine (clone 4G10), p85α (N-SH2, clone UB93–3), and p110β were purchased from Upstate Biotechnology/Chemicon/Millipore (Temecula, CA). The primary antibodies for IRS-1 and insulin receptor-β were purchased from Santa Cruz Biotechnology (Santa Cruz, CA). The primary antibody for β-actin was purchased from Novus Biologicals (Littleton, CO). Silica gel 60 TLC plates were purchased from EMD Biosciences (San Diego, CA). [γ-^32^P]ATP (sp. activity: 4,500 Ci/mmol) was purchased from MP Biomedicals (Solon, OH). All other chemicals were from Fisher Scientific (Fair Lawn, NJ) or Sigma Chemical (St. Louis, MO).

### Cell culture and treatments

The rat retinal ganglion cell line (RGC-5) was obtained as a gift from Dr. Neeraj Agarwal (University of North Texas Health Science Center, Fort Worth, Texas). RGC-5 cells (passages up to 10) were maintained in culture using Dulbecco’s Modified Eagle Medium (Invitrogen, Carlsbad, CA) along with 10% fetal bovine serum (HyClone, Logan, UT) and antibiotic and antimycotic solution (Sigma) in a humidified atmosphere of 95% air and 5% CO_2_, as described [12,31]. After the attainment of confluence, RGC-5 cells were trypsinized and seeded on to either 100 mm or 60 mm Petri dishes or 6 well plates. The subconfluent cells were then subjected to serum deprivation for 24 h, treated with 0.1 µM to 30 µM imatinib, and stimulated with either 30 ng/ml PDGF or 30 nM insulin for 6 min. Control and treated cells were washed with ice-cold phosphate buffered saline (PBS, HyClone), and lysed with laemmli sample buffer (for immunoblotting) or snap frozen using buffer A (for immunoprecipitation/in vitro kinase assays). Buffer A consisted of 50 mM Tris-HCl (pH 7.5), 0.1% Triton X-100, 1 mM EDTA, 1 mM EGTA, 50 mM sodium fluoride, 10 mM sodium β-glycerophosphate, 5 mM sodium pyrophosphate, 1 mM sodium orthovanadate, protease inhibitor cocktail (Sigma), 0.1% β-mercaptoethanol, and 1 µM LR-microcystin.

### Fluorescence and phase contrast microscopy

RGC-5 cells were seeded onto 6 well plates. The subconfluent cells were then maintained in complete medium (+FBS) or serum free medium (-FBS) for 48 h, during which time the cells were exposed to increasing concentrations of imatinib (1 µM to 30 µM). In addition, serum-free medium (-FBS) was supplemented with either 30 ng/ml PDGF or 30 nM insulin during RGC-5 cell exposure to imatinib. After 48 h, the cells were washed with PBS (pH 7.4), fixed with 4% paraformaldehyde for 15 min, and then labeled with 0.5 µg/ml 4,6 diamidino-2-phenylindole (DAPI). The apoptotic phenotype was then assessed by nuclear staining with DAPI (fluorescence microscopy) and by morphological changes (phase-contrast microscopy). For each treatment condition, at least 3 different fields were chosen to verify apoptotic cell death characterized by nuclear condensation, cytoplasmic shrinkage, and rounding-up of cells. The images of RGC-5 cells obtained from fluorescence and phase-contrast microscopy are representative of 4 to 5 separate experiments.

### Immunoblot analysis

RGC-5 cell lysates (10 µg protein each) were electrophoresed using pre-cast 4%–12% NuPage mini-gels (Invitrogen), and the resolved proteins were transferred to nitrocellulose membranes (Hybond C; Amersham Biosciences). The membranes were blocked and probed with the respective primary antibodies. The membranes were washed three times with Tris-buffered saline (TBS, pH 7.5). The components of TBS included 25 mM Tris base, 137 mM NaCl, 3 mM KCl, and 0.1% Tween-20. Subsequently, the immunoreactivity was detected using specific HRP-conjugated secondary antibodies followed by enhanced chemiluminescence (Amersham Biosciences), as described [12]. The protein bands were quantified using Biorad GS-800 calibrated densitometer.

### Immunoprecipitation of tyrosine phosphorylated proteins, IRS-1, p85α, or p110β subunits

RGC-5 lysates obtained using buffer A were sonicated (15 s×4) and centrifuged at 16,000x g (4 °C) for 10 min. The respective supernatants were then used for protein assays (Coomassie protein reagent, Pierce, Rockford, IL). The aliquots of supernatants (60 µg protein) were subjected to immunoprecipitation (4 °C, overnight) with 2 µg each of anti-phosphotyrosine, anti-IRS-1, anti-p85α, or anti-p110β primary antibody that was pre-conjugated (2 h at 4 °C) to Gammabind G Sepharose. Prior to the PI 3-kinase assays, the immunocomplexes were washed with buffer A and TNE buffer, which consisted of 10 mM Tris-HCl (pH 7.4), 150 mM NaCl, 5 mM EGTA, and 0.1 mM sodium orthovanadate [12].

### In vitro PI 3-kinase assays

PI 3-kinase assays were performed according to the following protocol [12]. After immunoprecipitation of proteins using specific primary antibodies, the respective immunocomplexes were subjected to PI 3-kinase assays by incubation at 35 °C for 10 min in the presence of 50 µl TNE buffer (pH 7.4), 20 µg/assay phosphatidylinositol substrate, and 10 µCi/assay γ-^32^P-ATP. The reactions were stopped by adding 20 µl of 6N HCl and 160 µl of CHCl_3_/CH_3_OH (1:1). Subsequently, the assay tubes were vortexed for 20 s and centrifuged at 16,000 xg at room temperature for 5 min. The phospholipid-containing lower organic phase from the respective reaction tubes was recovered and spotted on to silica gel thin layer chromatography plates that had been heated to 100 °C for approximately 1 h. The thin layer chromatography plates were then subjected to ascending chromatography using a freshly prepared solvent mixture that contained (in ml) 60 CHCl_3_; 47 CH_3_OH; 11.3 H_2_O; 2 NH_4_OH. Phosphatidylinositol 3-phosphate (PI3P) spots thus resolved were visualized and quantified by autoradiography and phosphorimager analyses (Molecular Dynamics, Sunnyvale, CA), respectively. As negative controls, mock immunoprecipitations were performed using lysis buffer, which revealed negligible formation of ^32^P-labeled PI3P.

### Statistical analyses

Results shown are the mean±standard error of the mean of 3 or more experiments. Statistical analyses of the data were performed by one-way repeated measures ANOVA followed by Bonferroni *t*-test. Values of p<0.05 were considered statistically significant.

## Results

### Imatinib induces RGC apoptosis with or without PDGF/insulin exposure

**Figure 1:**
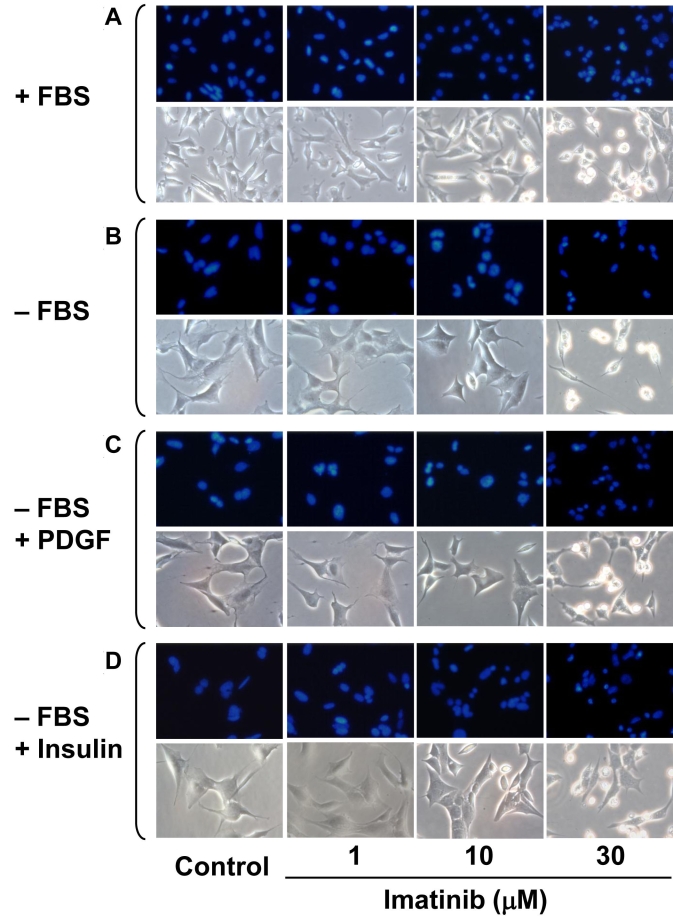
Concentration dependency for imatinib-induced phenotypic changes in RGC-5 cells. Subconfluent RGC-5 cells were maintained in complete medium (+FBS) or serum-free medium (-FBS) for 48 h, during which time the cells were exposed to increasing concentrations of imatinib (1 µM to 30 µM; **A, B**). In addition, serum-free medium (-FBS) was supplemented with either 30 ng/ml PDGF or 30 nM insulin during RGC-5 cell exposure to imatinib (**C, D**). The apoptotic phenotype was then assessed by nuclear DAPI staining (fluorescence microscopy) and morphological changes (phase-contrast microscopy). The images shown are representative of 4 to 5 separate experiments.

Previously, we and several other investigators showed that PDGF or insulin promotes the survival of neuronal cells, including RGCs [3,4,12]. In the present study, we examined the effects of imatinib, a PDGF receptor tyrosine kinase inhibitor, on the phenotypic changes in RGC-5 cells. Initial experiments involved the maintenance of subconfluent RGC-5 cells in complete medium (+FBS) for 48 h, during which time the cells were exposed to increasing concentrations of imatinib (1 µM to 30 µM) and then examined for phenotypic changes. Figure 1A shows that in comparison with control, imatinib treatments with 10 to 30 µM concentrations for 48 h caused progressive increases in nuclear condensation as revealed by nuclear staining with DAPI (fluorescence microscopy) and cytoplasmic shrinkage and rounding up of cells as revealed by phase-contrast microscopy. In addition, imatinib produced similar phenotypic changes in RGC-5 cells maintained under serum-free conditions in the absence (Figure 1B) or presence of PDGF (Figure 1C), or insulin (Figure 1D). These data suggest that PDGF receptor tyrosine kinase inhibition by imatinib has the potential to induce RGC apoptosis, and that supplementation with neurotrophic factors such as PDGF or insulin under these conditions may not rescue apoptotic cell death.

### Imatinib induces caspase-3/PARP cleavage and diminishes Akt phosphorylation in RGCs

**Figure 2:**
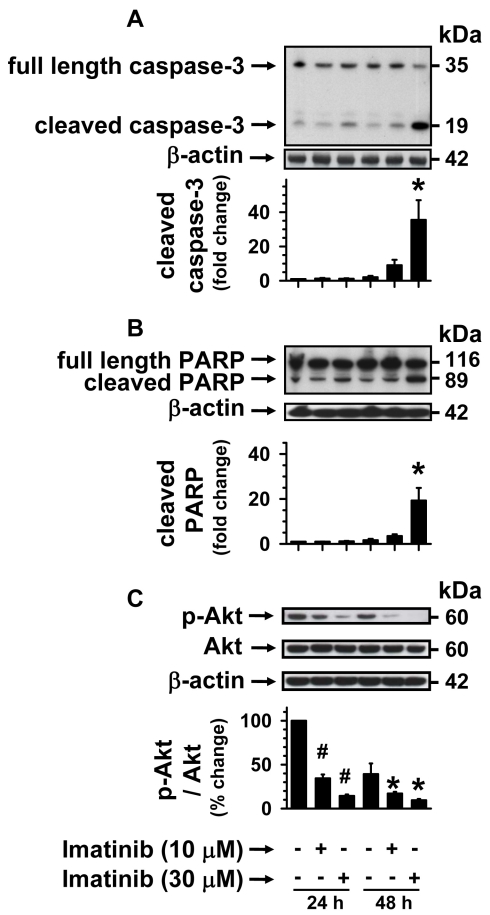
Concentration-dependent effects of imatinib on caspase-3/PARP cleavage and Akt phosphorylation. Subconfluent RGC-5 cells were maintained in serum free medium (-FBS) for 24 h or 48 h, during which time the cells were exposed to increasing concentrations of imatinib (10 µM to 30 µM). The cell lysates were subjected to immunoblot analysis using primary antibodies specific for **A**) caspase-3 that detects full-length and cleaved caspase-3, and **B**) PARP that detects full-length PARP and cleaved PARP, and **C**) phospho-Akt(Ser473) and total Akt. To normalize the expression of cleaved caspase-3 and cleaved PARP and the phosphorylation of Akt, we used the housekeeping gene β-actin as an internal control. The respective bar graphs shown are the mean±SEM values from 3 to 5 experiments. The asterisk and the sharp (hash mark) indicate a p<0.05 compared with the respective controls.

Apoptotic cell death is induced by the upregulation of cleaved caspase-3 and cleaved PARP. Cleaved caspase-3 is the activated form of cytosolic caspase-3, whereas nuclear PARP is one of the substrates of activated caspase-3. The activation of caspase-3 may result from diminution in the phosphorylation or activation of cell survival kinases, including Akt, and so we examined if imatinib perturbs these pathways in RGC-5 cells. Subconfluent RGC-5 cells were maintained in serum-free conditions for 24 h or 48 h, during which time the cells were exposed to 10 µM and 30 µM imatinib. The cell lysates were subjected to immunoblotting using the following primary antibodies: anti-caspase-3 primary antibody that detects endogenous full length inactive caspase-3 (35 kDa) and the cleaved caspase-3 (19 kDa; Figure 2A); and anti-PARP primary antibody that detects endogenous full length PARP (116 kDa) and the catalytic domain-containing cleaved fragment (89 kDa; Figure 2B). Figure 2A shows that in comparison with control, imatinib treatments at 10 µM and 30 µM concentrations for 24 h did not produce significant increases in cleaved caspase-3 expression. However, when the incubation time was prolonged to 48 h, there were progressive increases in the upregulation of cleaved caspase-3. As shown in Figure 2A, 10 µM and 30 µM imatinib caused significant increases in the expression levels of cleaved caspase-3 by approximately 4.4 fold and 17.3 fold, respectively, compared with the respective control at the 48 h time point. In a similar manner, imatinib treatments at 10 µM and 30 µM concentrations for 48 h caused progressive increases in the expression levels of cleaved PARP (Figure 2B). The graph shows that 30 µM imatinib increased the expression of cleaved PARP by roughly 8.7 fold, compared with the respective control at the 48 h time point. The upregulation of cleaved caspase-3 and cleaved PARP was preceded by sustained diminutions in Akt Ser473 phosphorylation, as can be seen with 10 µM and 30 µM imatinib treatments for 24 h or 48 h (Figure 2C). These data suggest that inhibition of PDGF receptor tyrosine kinase by imatinib may suppress the activation of lipid kinase (PI 3-kinase) upstream of Akt and the phosphorylation of protein kinases downstream of PI 3-kinase. It is therefore important to examine whether imatinib induces RGC apoptosis by dysregulating agonist-specific activation of lipid kinase and protein kinase survival signaling.

### Acute imatinib treatment inhibits PDGF but not insulin receptor phosphorylation

**Figure 3:**
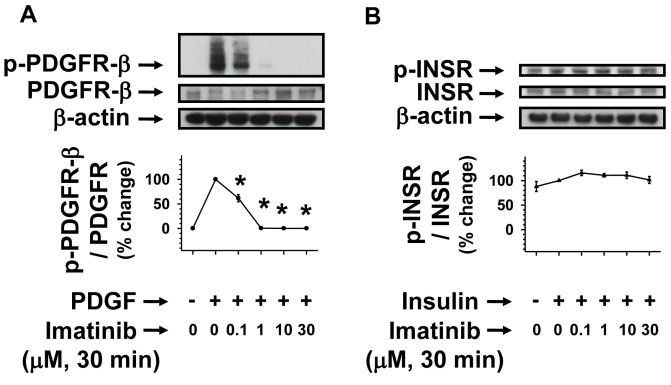
Concentration-dependent effects of imatinib on PDGF-induced versus insulin-induced receptor phosphorylation. Serum-deprived (24 h) RGC-5 cells were pretreated without (control) or with increasing concentrations of imatinib (0.1 µM to 30 µM) for 30 min. Subsequently, control and imatinib-treated cells were stimulated with either 30 ng/ml PDGF or 30 nM insulin for 6 min. The cell lysates were subjected to immunoblot analysis for receptor tyrosine phosphorylation (**A, B**) using the indicated primary antibodies (See Methods). To normalize the changes in protein phosphorylation in the immunoblots, β-actin was used as an internal control. Note: For data analyses, PDGF- or insulin-induced receptor phosphorylation in the absence of imatinib was normalized to 100%. The respective linear graphs shown are the mean±SEM values from 3 experiments. The asterisk denotes a p<0.05 compared with the respective PDGF-induced receptor phosphorylation.

To determine whether imatinib dysregulates agonist-specific receptor tyrosine phosphorylation, we subjected serum-deprived (24 h) RGC-5 cells to pretreatments without (control) or with imatinib (0.1 to 30 µM) for 30 min. Subsequently, control and imatinib-pretreated cells were stimulated with PDGF or insulin for 6 min. As shown in Figure 3A, PDGF stimulation of RGC-5 cells resulted in robust increases in PDGFR tyrosine phosphorylation compared with control, and pretreatment of RGC-5 cells with increasing concentrations of imatinib attenuated PDGF-induced PDGFR tyrosine phosphorylation. The extent of decreases in PDGF-induced PDGFR tyrosine phosphorylation with imatinib at 0.1, 1, 10, and 30 µM concentrations were 47%, 96%, 99%, and 99%, respectively (p<0.05). Figure 3B shows that insulin receptors are tyrosine phosphorylated in RGC-5 cells under basal conditions, characteristic of constitutively active insulin receptors in the rodent retinas [10,32]. Notably, imatinib did not inhibit insulin-induced insulin receptor tyrosine phosphorylation at any of the concentrations used in this study (Figure 3B). These data support the specificity of imatinib toward inhibiting PDGFR tyrosine kinase and the lack of its inhibitory effects on insulin receptor tyrosine kinase activity.

### Acute imatinib treatment inhibits PDGF-induced but not insulin-induced PI 3-kinase

**Figure 4:**
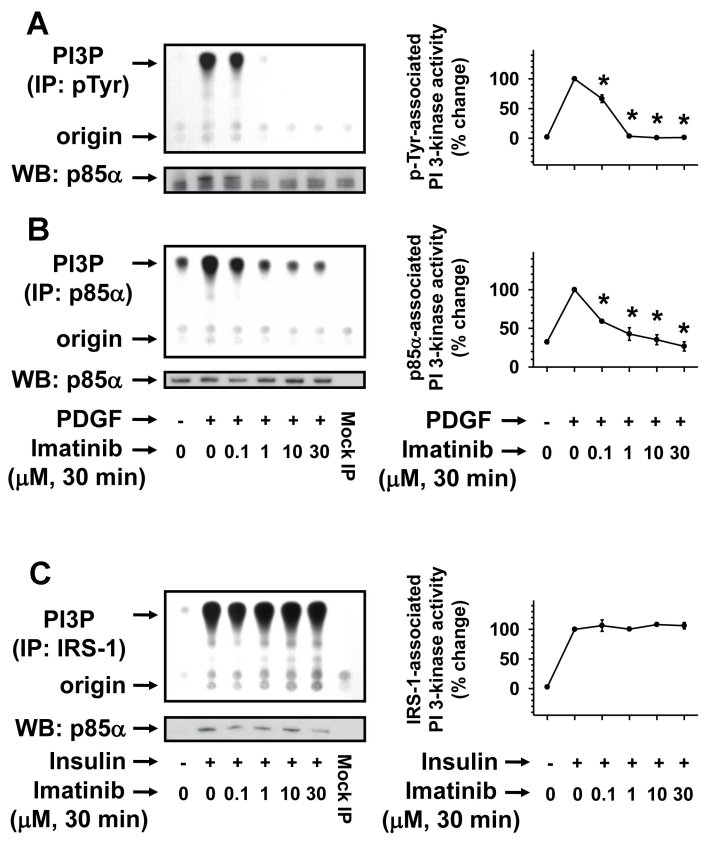
Concentration-dependent effects of imatinib on PDGF-induced versus insulin-induced PI 3-kinase. Serum-deprived (24 h) RGC-5 cells were pretreated without (control) or with increasing concentrations of imatinib (0.1 µM to 30 µM) for 30 min. Subsequently, control and imatinib-treated cells were stimulated with either 30 ng/ml PDGF or 30 nM insulin for 6 min. The cell lysates were subjected to immunoprecipitation followed by PI 3-kinase assays or immunoblot analysis (**A-C**) using the indicated primary antibodies (see Methods). The representative thin layer chromatogram and the immunoblots for imatinib regulation of PDGF-induced PI 3-kinase activity (**A, B**) and insulin-induced PI 3-kinase activity (**C**) are shown. Note: For data analyses, PDGF- or insulin-induced PI 3-kinase activity in the absence of imatinib was normalized to 100%. The respective linear graphs shown are the mean±SEM values from 3 experiments. The asterisk indicates a p<0.05 compared with the respective PDGF-induced PI 3-kinase activity.

Previous studies have shown that imatinib inhibits PDGF-induced Akt phosphorylation in vascular smooth muscle cells [33] and IRS-associated PI 3-kinase activity in leukemia cells [34,35]. These findings suggest the potential for imatinib to inhibit PDGF receptor-associated PI 3-kinase as well as IRS-associated PI 3-kinase activity in a cell type-specific manner. In the present study, we therefore examined the effects of imatinib on PDGF- and insulin-induced PI 3-kinase activity in RGC-5 cells. As shown in Figure 4A,B, PDGF stimulation of RGC-5 cells for 6 min resulted in robust increases in phosphotyrosine-associated (Figure 4A) and p85α-associated PI 3-kinase activity (Figure 4B), which was inhibited by pretreatments with increasing concentrations of imatinib for 30 min. The diminished PI 3-kinase activity was due to imatinib inhibition of p85α recruitment by PDGF (Figure 4A) without affecting the expression of p85α (Figure 4B). At 10 µM imatinib concentration, PDGF-induced phosphotyrosine- and p85α-associated PI 3-kinase activity declined to values comparable to the basal PI 3-kinase activity. In contrast, imatinib did not inhibit insulin-induced IRS-1 association with p85α or IRS-1-associated PI 3-kinase activity at any of the concentrations used in this study (Figure 4C). These data further support the specificity of imatinib action in that it inhibits PDGF receptor tyrosine phosphorylation to inhibit p85α recruitment and consequently attenuates phosphotyrosine- and p85α- associated PI 3-kinase activity. In parallel, imatinib did not alter insulin-induced insulin receptor tyrosine phosphorylation, p85α recruitment to IRS-1, and IRS-1 associated PI 3-kinase activity in RGC-5 cells.

### Acute imatinib treatment inhibits PDGF-induced but not insulin-induced Akt/GSK-3β/p70S6kinase phosphorylation

**Figure 5:**
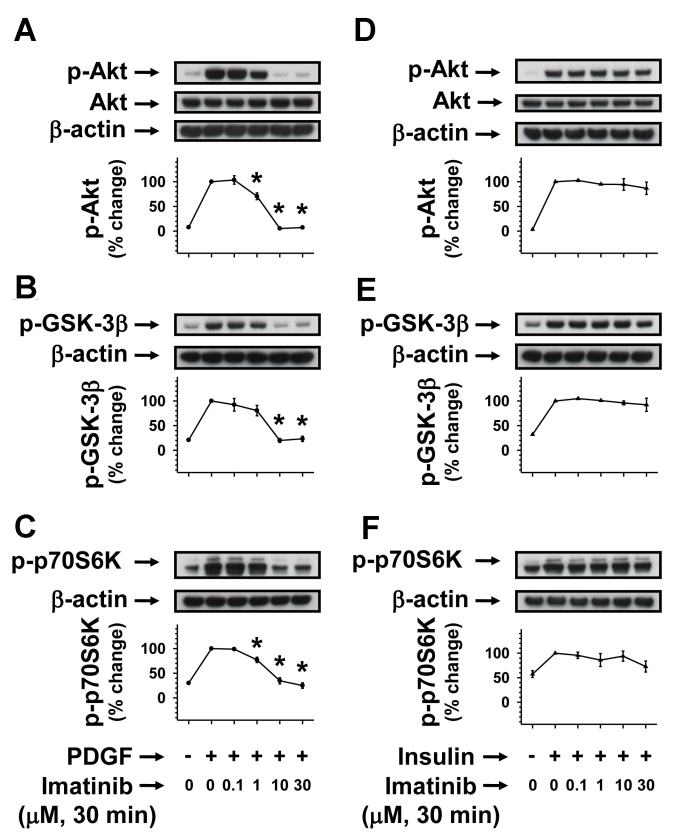
Concentration-dependent effects of imatinib on PDGF-induced versus insulin-induced Akt/GSK-3β/p70S6kinase phosphorylation. Serum-deprived (24 h) RGC-5 cells were pretreated without (control) or with increasing concentrations of imatinib (0.1 µM to 30 µM) for 30 min. Subsequently, control and imatinib-treated cells were stimulated with either 30 ng/ml PDGF or 30 nM insulin for 6 min. The cell lysates were subjected to immunoblot analysis for Akt, GSK-3β, and p70S6 kinase phosphorylation (**A-C**, and **D-F**) using the indicated primary antibodies (see Methods). To normalize the changes in protein phosphorylation in the immunoblots, we used β-actin as an internal control. Note: For data analyses, PDGF- or insulin-induced protein kinase phosphorylation in the absence of imatinib was normalized to 100%. The respective linear graphs shown are the mean±SEM values from 3 experiments. The asterisk indicates a p<0.05 compared with the respective PDGF-induced protein kinase phosphorylation..

The data shown in Figure 3 and Figure 4 suggest that imatinib may cause selective inhibition of PDGF-induced PI 3-kinase downstream signaling events, including Akt, GSK-3β, and p70S6kinase phosphorylation without affecting insulin-induced Akt signaling. Recent studies with hepatoma cells have shown that imatinib inhibits c-Abl cytosolic tyrosine kinase to attenuate insulin-induced but not IGF-1-induced phosphorylation of Akt and GSK-3β [26]. Hence, it is important to examine the selectivity of imatinib toward inhibiting agonist-specific signaling events even at the level of protein phosphorylation downstream of PI 3-kinase. In the present study, acute imatinib pretreatment caused progressive decreases in PDGF-induced (Figure 5A-C) but not insulin-induced (Figure 5D-F) phosphorylation of Akt, GSK-3β, and p70S6kinase. These data suggest that c-Abl may not be a likely intermediary component for insulin-induced Akt signaling events in RGC-5 cells.

### Prolonged imatinib treatment inhibits PDGF but not insulin receptor phosphorylation

**Figure 6:**
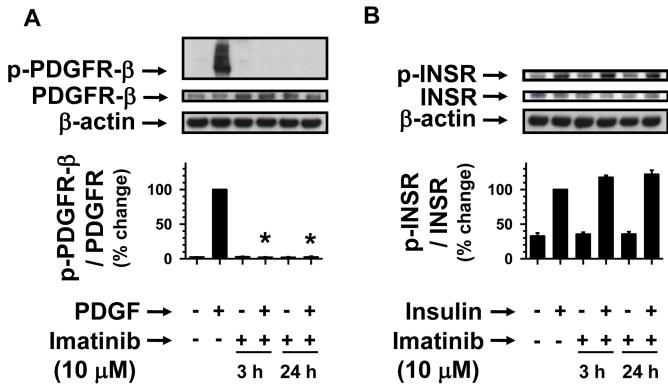
Time-dependent effects of imatinib on PDGF-induced versus insulin-induced receptor phosphorylation. Serum-deprived (24 h) RGC-5 cells were pretreated without (control) or with 10 µM imatinib for increasing time intervals (3 h and 24 h). Subsequently, control and imatinib-treated cells were stimulated with either 30 ng/ml PDGF or 30 nM insulin for 6 min. The cell lysates were subjected to immunoblot analysis for receptor tyrosine phosphorylation (**A,B**) using the indicated primary antibodies (See Methods). To normalize the changes in protein phosphorylation in the immunoblots, we used β-actin as an internal control. Note: For data analyses, PDGF- or insulin-induced receptor phosphorylation in the absence of imatinib was normalized to 100%. The respective bar graphs shown are the mean±SEM values from 3 to 4 experiments. The asterisk indicates a p<0.05 compared with the respective PDGF-induced receptor phosphorylation.

To determine the sustained effects of imatinib that dysregulates agonist-specific receptor tyrosine phosphorylation, we subjected serum-deprived (24 h) RGC-5 cells to pretreatments without (control) or with 10 µM imatinib for 3 h and 24 h. Subsequently, control and imatinib-pretreated cells were stimulated with PDGF or insulin for 6 min. As shown in Figure 6A, imatinib pretreatments for 3 h and 24 h completely abolished PDGF-induced PDGF receptor tyrosine phosphorylation. In contrast, even after prolonged incubation with imatinib for 3 h and 24 h, there were no decreases in insulin-induced insulin receptor tyrosine phosphorylation (Figure 6B). Thus, the data shown in Figure 3A,B and Figure 6A,B indicate that acute or chronic exposure to imatinib maintains its specificity toward inhibiting PDGF receptor tyrosine kinase.

### Prolonged imatinib treatment inhibits PDGF-induced but not insulin-induced PI 3-kinase

**Figure 7:**
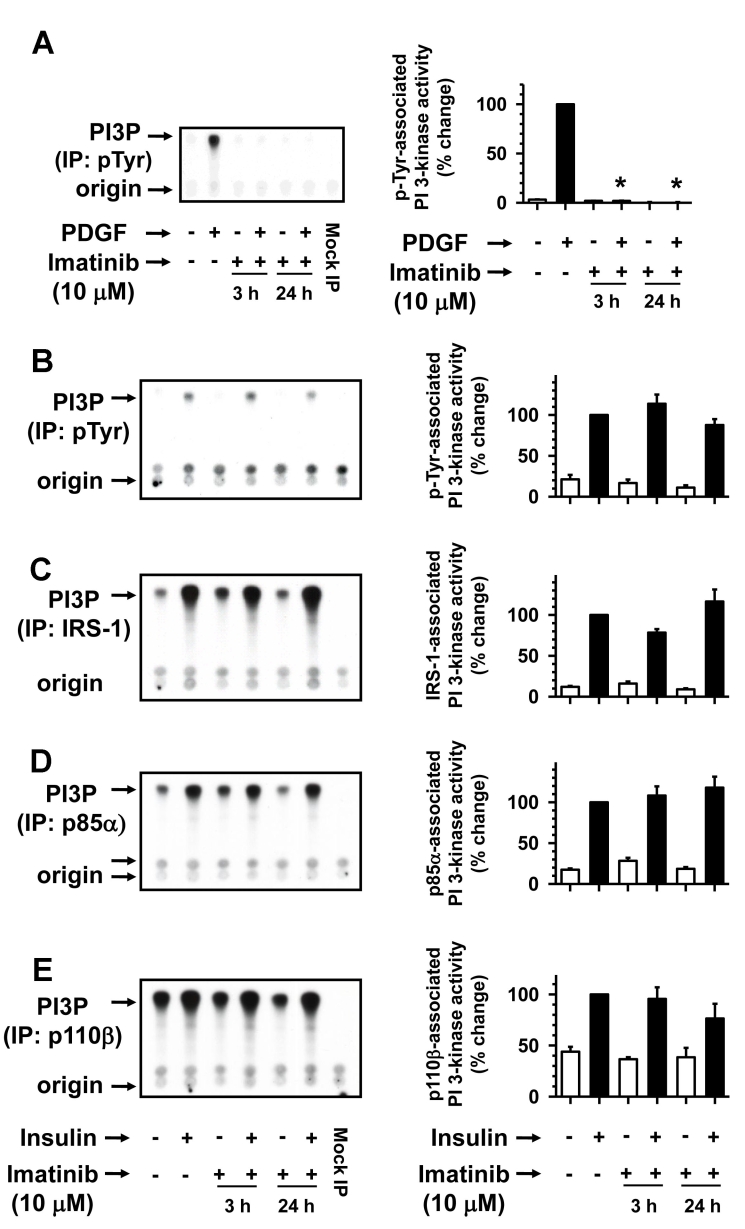
Time-dependent effects of imatinib on PDGF-induced versus insulin-induced PI 3-kinase. Serum-deprived (24 h) RGC-5 cells were pretreated without (control) or with 10 µM imatinib for increasing time intervals (3 h and 24 h). Subsequently, control and imatinib-treated cells were stimulated with either 30 ng/ml PDGF or 30 nM insulin for 6 min. The cell lysates were subjected to immunoprecipitation followed by PI 3-kinase assays or immunoblot analysis (**A** and **B-E**) using the indicated primary antibodies (see Methods). The representative thin layer chromatogram and the immunoblots for imatinib regulation of PDGF-induced PI 3-kinase activity (**A**) and insulin-induced PI 3-kinase activity (**B-E**) are shown. Note: For data analyses, PDGF- or insulin-induced PI 3-kinase activity in the absence of imatinib was normalized to 100%. The respective bar graphs shown are the mean±SEM values from 3 to 4 experiments. The asterisk indicates a p<0.05 compared with the respective PDGF-induced PI 3-kinase activity.

Recent studies demonstrate that prolonged incubation of leukemia cells with imatinib results in the generation of endogenous ceramides [36], which may mediate apoptotic cell death by inhibiting PDGF- and insulin-induced PI 3-kinase activity [37]. In this regard, treatment of rat retinal neuronal cells with C_2_-ceramide inhibits insulin-induced IRS-associated PI 3-kinase activity (unpublished observations). To verify whether long-term imatinib exposure affects PDGF as well as insulin-induced PI 3-kinase activity, further studies were performed. As shown in Figure 7A, imatinib pretreatments for 3 h and 24 h completely abolished PDGF-induced phosphotyrosine-associated PI 3-kinase activity. In contrast, imatinib pretreatments for 3 h and 24 h did not cause significant changes in insulin-induced PI 3-kinase activity associated with phosphotyrosine, IRS-1, p85α, and p110β immunocomplexes (Figure 7B-E). Thus, assessment of insulin-induced PI 3-kinase activity at the level of tyrosine phosphorylated proteins, IRS-1 adaptor molecule, p85 regulatory, and p110 catalytic subunits reveals that insulin-induced PI 3-kinase activation is refractory to imatinib treatment. Overall, the present findings support the specificity of long-term imatinib treatment in inhibiting PDGF receptor-mediated PI 3-kinase activation while maintaining insulin receptor-mediated IRS signaling in RGC-5 cells.

### Prolonged imatinib treatment inhibits PDGF-induced but not insulin-induced Akt/GSK-3β/p70S6kinase phosphorylation

**Figure 8:**
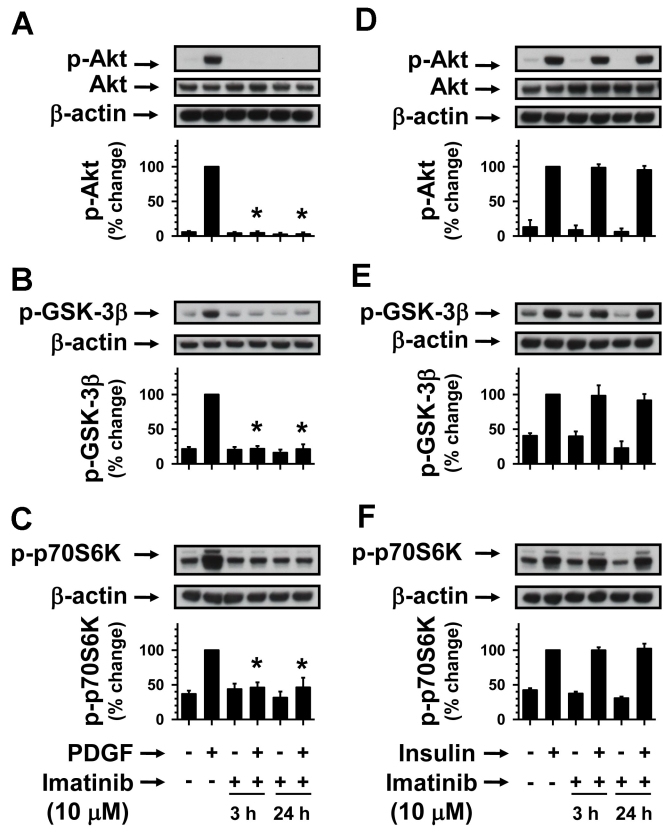
Time-dependent effects of imatinib on PDGF-induced versus insulin-induced Akt/GSK-3β/p70S6kinase phosphorylation. Serum-deprived (24 h) RGC-5 cells were pretreated without (control) or with 10 µM imatinib for increasing time intervals (3 h and 24 h). Subsequently, control and imatinib-treated cells were stimulated with either 30 ng/ml PDGF or 30 nM insulin for 6 min. The cell lysates were subjected to immunoblot analysis for Akt, GSK-3β, and p70S6kinase phosphorylation (**A-C**, and **D-F**) using the indicated primary antibodies (see Methods). To normalize the changes in protein phosphorylation in the immunoblots, we used β-actin as an internal control. Note: For data analyses, PDGF- or insulin-induced protein kinase phosphorylation in the absence of imatinib was normalized to 100%. The respective bar graphs shown are the mean±SEM values from 3 to 4 experiments. The asterisk indicates a p<0.05 compared with the respective PDGF-induced protein kinase phosphorylation.

To further support the observations shown in Figure 6 and Figure 7, we examined the effects of long-term imatinib exposure under similar conditions. As shown in Figure 8A-C, imatinib pretreatments for 3 h and 24 h completely abolished PDGF-induced phosphorylation of Akt, GSK-3β, and p70S6kinase, but not insulin-induced phosphorylation of Akt, GSK-3β, and p70S6kinase (Figure 8D-F). Together, the present study demonstrates that acute or chronic exposure to imatinib inhibits PDGF-induced PI 3-kinase/Akt signaling but maintains an apparent insulin-induced PI 3-kinase/Akt prosurvival signaling.

## Discussion

**Figure 9:**
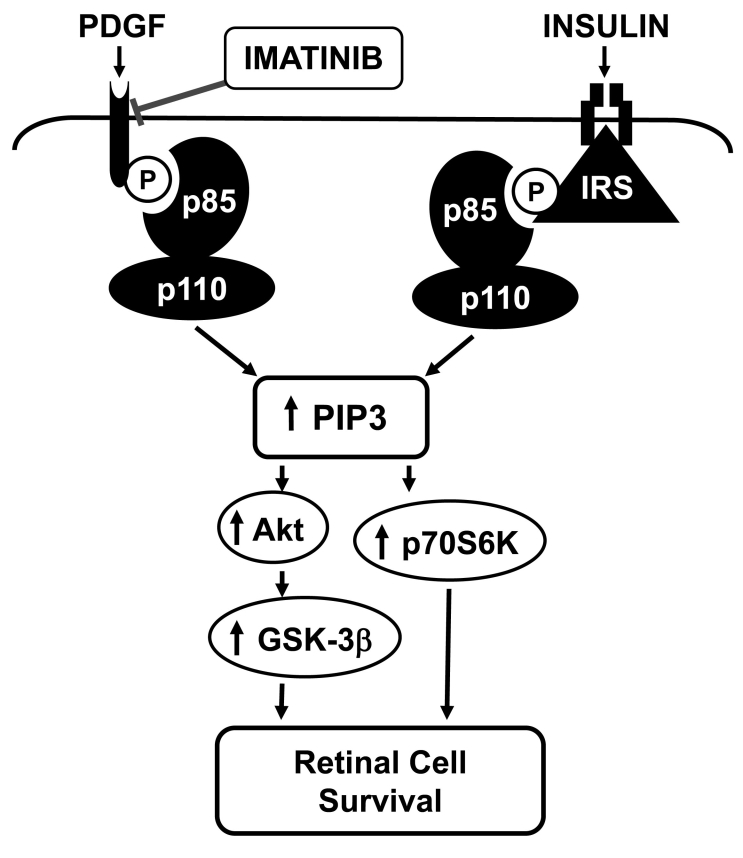
Schematic showing imatinib dysregulation of PI 3-kinase/Akt signaling in RGCs. PI 3-kinase is a heterodimer consisting of p85 regulatory and p110 catalytic subunits. Imatinib inhibition of PDGFR tyrosine kinase abrogates PDGF-induced PDGFR tyrosine phosphorylation, p85 regulatory subunit recruitment, PI 3-kinase activity, and the phosphorylation of downstream effectors such as Akt, GSK-3β, and p70S6kinase. In contrast, imatinib exposure maintains insulin receptor-mediated IRS-associated PI 3-kinase activity and the downstream phosphorylation of Akt, GSK-3β, and p70S6kinase. Thus, an imbalance between receptor- and IRS-associated PI 3-kinase activity attenuates coordinated increases in phosphatidylinositol 3,4,5-trisphosphate (PIP3) lipids. The resultant diminutions in the overall phosphorylation of Akt, GSK-3β, and p70S6kinase increase the propensity toward apoptotic cell death.

The present study provides evidence that the survival of RGCs, a key component of the central nervous system, is compromised by impaired PDGF receptor signaling. Imatinib inhibition of PDGF receptor signaling leads to apoptotic cell death, which is not rescued by insulin that triggers prosurvival signaling pathway (Figure 9). The major findings of the present study are as follows: 1) RGCs that possess functional PDGF receptors and insulin receptors undergo apoptosis characterized by upregulation of cleaved caspase-3 and PARP after exposure to imatinib, an inhibitor of PDGF receptor tyrosine kinase; 2) the apoptotic phenotype resulting from inhibition of PDGF receptor tyrosine kinase is not rescued by insulin, a neuroprotective agonist; 3) specifically, the apoptotic effect of imatinib is preceded by early and sustained inhibition of PDGF-induced signaling events such as PDGF receptor tyrosine phosphorylation, phosphotyrosine- and p85α-associated PI 3-kinase activity, and downstream phosphorylation of Akt, GSK-3β, and p70S6kinase; and 4) imatinib does not affect insulin-induced signaling events such as IRS- and p85α-associated PI 3-kinase activity and downstream phosphorylation of Akt, GSK-3β, and p70S6kinase, which, however, do not prevent imatinib-induced apoptosis. Together, these data suggest that an imbalance in PI 3-kinase activation resulting from impaired PDGF receptor-mediated p85α recruitment and normal IRS-mediated p85α recruitment may compromise the coordinated increases in intracellular PIP3 lipid species, which are critical for neuronal cell survival [30].

PI 3-kinase activation and PIP3 production are critical for neuronal cell survival and development, neurite formation and elongation, and axon specification [38-40]. This effect has been demonstrated in several neuronal cell types, including RGCs [41], hippocampal neurons [38,42,43], cerebellar granule neurons [43], and PC12 cells [44]. Overexpression or microinjection of constitutively active PI 3-kinase/Akt promotes neurite outgrowth and neuronal cell survival [45-47], whereas wortmannin inhibition of PI 3-kinase/Akt inhibits neurite outgrowth and induces neuronal cell apoptosis [4,44]. The present observations from imatinib-treated RGCs raise the possibility that an imbalance in IRS-dependent and IRS-independent PI 3-kinase signaling pathways may dysregulate neuronal cell phenotype by inhibiting neurite and axon formation and inducing apoptosis.

Several studies have shown that IRS-dependent and IRS-independent PI 3-kinase/Akt signaling pathways mediate the survival of neuronal cells. In this regard, insulin and insulin-like growth factor-1 utilize IRS as the intermediary signaling component to activate PI 3-kinase/Akt survival signaling [11,40]. In contrast, growth factors (e.g., PDGF and hepatocyte growth factor) and neurotrophins (e.g., brain-derived neurotrophic factor, neurotrophin-3, and nerve growth factor) utilize IRS-independent mechanisms to activate PI 3-kinase/Akt survival signaling [12,14,48-50]. Thus, several neurotrophic factors promote the survival of neuronal cells by distinct activation of IRS-dependent or IRS-independent PI 3-kinase, which results in the formation of PIP3 species and downstream phosphorylation of protein kinases such as Akt, GSK-3β, and p70S6kinase. The present study has employed a neuronal cell model system that directly compares IRS-dependent and IRS-independent PI 3-kinase/Akt survival signaling after stimulation with insulin and PDGF, respectively. Our observations provide the first direct evidence that imatinib disruption of PDGF-induced IRS-independent PI 3-kinase/Akt signaling is associated with the induction of apoptosis, which is not prevented by apparent increases in insulin-induced IRS-dependent PI 3-kinase/Akt signaling events. The inability of insulin to exhibit neuroprotective effects under these conditions strongly suggests that PI 3-kinase/Akt signaling events via IRS-dependent and IRS-independent mechanisms are not redundant but independent pathways for optimal survival of neuronal cells.

At this juncture, it is pertinent to note that imatinib therapy in CML patients with diabetes improves insulin sensitivity and fasting blood glucose levels [24,25]. On the contrary, recent studies with hepatoma cells have shown that imatinib attenuates insulin-induced phosphorylation of Akt and glycogen synthase kinase-3β (GSK-3β) [26]. In the present study, imatinib treatment in RGCs maintains insulin-induced PI 3-kinase/Akt signaling, which, however, is not sufficient to prevent apoptotic cell death in the context of abrogated PDGF receptor signaling. Future studies are clearly warranted, and should determine the extent to which systemic and ocular infusion of imatinib in rodent models affects PDGF *vs* insulin-induced pro-survival signaling in the neuroretina.

PDGF exerts neuroprotective effects in several neuronal cell types. After optic nerve axotomy, RGCs undergo apoptosis due to diminished PDGF expression levels [16]. In hippocampal neurons, PDGF protects against metabolic insults resulting from energy deprivation and oxidative insults arising from hydroxyl radical exposure [51]. In these neurons, PDGF also inhibits N-methyl-D-aspartate (NMDA) receptor-mediated excitotoxicity [52]. Notably, mouse brains deficient in neuronal PDGF receptor-β are vulnerable to cerebral damage, as revealed by increased neuronal cell death by NMDA [18]. In glioma cells, PDGF-induced PI 3-kinase signaling enhances cell surface expression of glutamate transporters to promote glutamate transport activity, thereby attenuating cytotoxicity arising from extracellular glutamate [14]. In brainstem neurons, PDGF-induced PI 3-kinase/Akt signaling prevents hypoxia-induced apoptosis [15,53]. In neuroblastoma cells, PDGF prevents gp120-induced cytochrome C release and apoptosis [50]. Thus, PDGF promotes neuronal cell survival by preventing aberrant processes that contribute to excitotoxic or apoptotic cell death. Our present findings with RGC-5 cells demonstrate that imatinib disruption of PDGF receptor signaling is associated with induction of apoptosis, which is not prevented by insulin. Together, these data suggest that PDGF- and insulin-induced PI 3-kinase/Akt signaling may differentially regulate diverse cellular processes that are critical for neuronal survival.

RGC-5 cells express several ganglion cell markers including Thy-1 and NMDA receptors, and thereby possess the characteristics of primary RGCs in culture [31,54]. In addition, RGC-5 cells exhibit glial-like undifferentiated phenotype [55]. Recent studies demonstrate that treatment of RGC-5 cells with staurosporine, a broad-spectrum protein kinase inhibitor, promotes transition of undifferentiated RGC-5 cells to a differentiated phenotype without inducing apoptosis [55]. Of importance, PDGF receptor-deficient neuroprogenitor cells exhibit increased apoptosis and reduced capacity to differentiate in response to PDGF, suggesting the importance of intact PDGF receptor signaling for neuronal cell survival and differentiation [56]. Our present observations with imatinib-treated RGC-5 cells suggest that the activation of PDGF receptor tyrosine kinase is critical for retinal neuronal cell survival.

PDGF receptor antagonism induces apoptotic loss of RGCs (present study), neuroprogenitor cells [56], and brainstem neurons [15]. In addition, imatinib inhibition of PDGF receptor signaling is implicated in the development of retinal edema [19-23] and cerebral edema [57]. The current observations of imatinib-induced apoptosis in retinal cell culture model may provide a possible explanation for these observations but further studies are warranted. In conclusion, the present findings strongly suggest that intact PDGF receptor signaling characterized by IRS-independent activation of PI 3-kinase/Akt is critically important for retinal neuronal cell survival.
